# Serum ferritin and iron deficiency in adolescent schoolchildren with depression in northern Sudan: unmatched case-control study

**DOI:** 10.3389/fpsyt.2025.1622027

**Published:** 2025-07-22

**Authors:** Ahmed A. Hassan, Hatim Y. Alharbi, Ishag Adam

**Affiliations:** ^1^ Faculty of Medicine, University of Khartoum, Khartoum, Sudan; ^2^ Department of Psychiatry, College of Medicine, Qassim University, Buraydah, Qassim, Saudi Arabia; ^3^ Department of Obstetrics and Gynecology, Qassim University, Buraydah, Qassim, Saudi Arabia

**Keywords:** depression, serum ferritin, adolescents, age, female, iron deficiency

## Abstract

**Background:**

The direction of the association between depression and serum ferritin among adolescents needs to be explored further. There is no data on the association between serum ferritin and depression in Sudan. Therefore, the current study aimed to compare serum ferritin and iron deficiency between adolescents with depression and healthy controls in northern Sudan.

**Methods:**

An unmatched case-control study was conducted among adolescent school children in River Nile State, Sudan. The cases were adolescents with depression (n = 61), and an equal number of healthy adolescents were controls. The Patient Health Questionnaire (PHQ−9) was used to assess depression. Multivariate regression analysis was performed.

**Results:**

The median serum ferritin level (6.3 [IQR 2.9–13.8] μg/l vs. 25.4 [IQR 19.5–37.1] μg/l, p <0.001) was significantly lower in adolescents with depression than in healthy controls. A significant negative correlation was found between the depression scale and serum ferritin levels (r = -0.596, p < 0.001). In multivariate analysis, while increasing age (adjusted odds ratio [AOR] = 1.44, 95% confidence interval [CI] 1.05–1.98), being female (AOR = 11.19, 95% CI 4.17–29.99), and iron deficiency (AOR = 76.87, 95% CI 9.08–650.0) were positively associated with depression, serum ferritin was negatively associated with depression (AOR = 0.96, 95% CI 0.94–0.99).

**Conclusion:**

This study revealed a notable negative association between serum ferritin levels and depression. Adolescents with depression should be assessed for serum ferritin and iron deficiency.

## Introduction

Mental health disorders, including depression, are among the leading causes of illness and disability among adolescents (1). There is an increasing trend of adolescent mental health disorders, such as depression, at the global level, including sub-Saharan Africa (2), where the heavy burden of adolescents’ mental health disorders, including depression, is shouldered (3). In sub-Saharan Africa, it is estimated that more than one-quarter (26.9%) of adolescents have depression (3). Several factors, such as age (4), being female (5), tobacco use (5, 6), obesity (7–9), and the level of maternal education (10), have been found to be associated with depression among adolescents.

On the other hand, globally, iron deficiency is the most prevalent nutritional disorder, particularly in low- and middle-income countries (11, 12). Research has shown that a poor diet contributes to the genesis and course of depression, and vice versa (13–15). Recently, there has been more concern regarding the association between serum ferritin and iron deficiency and mental health disorders, including depression, among different populations, including adolescents (9, 16–20). A positive association between depression and decreased ferritin levels in adolescents and young adults has been reported (17, 21). On the other hand, iron deficiency has been reported to be associated with poorer mental health, including depression, in adolescents (16). While the association between serum ferritin, iron deficiency, and depression among adolescents has been studied in many countries (9, 16–21), no such data exist in Sudan. Mental health disorders, including depression, and nutritional deficiencies, including iron deficiency, among adolescents are both public health problems in Sudan [23–25], which is similar to African contexts (12, 22). Studying serum ferritin and iron deficiency in adolescents with depression in northern Sudan is essential for understanding the potential links between nutritional status and mental health. Such research can inform targeted interventions, improve adolescent health outcomes, and contribute to a broader understanding of how dietary factors influence psychological well-being in vulnerable populations, such as adolescents. Therefore, this study aims to compare serum ferritin and iron deficiency between adolescents with depression and healthy controls in northern Sudan.

## Methods

### Study design and setting

This was an unmatched case-control study conducted among adolescent schoolchildren (61 with depression and 61 healthy controls) at government schools in the locality of Almatamah, northern Sudan, from August to September 2022. Almatamah is located in the River Nile State in northern Sudan, approximately 100 kilometers from Khartoum, the capital of Sudan.

### Research question

Is there a difference in serum ferritin levels and iron deficiency between adolescents with depression and healthy controls in northern Sudan?

### Case and control definitions

In this study, the strengthening of the reporting of observational studies in epidemiology (STROBE) guidelines were strictly followed (23). Adolescents were considered depressed when their Patient Health Questionnaire (PHQ-9) scores were more than 4; any adolescent with a PHQ-9 score of four or less was considered a control. More details are provided in our previously published work regarding the PHQ-9 scores for depression (24).

### Sampling technique

In Almatamah locality, there are three districts, among which is Wad Hamid district. In Wad Hamid district, there are 16 government schools, each serving male or female students. Six schools (three for each sex) were randomly selected using simple random sampling via a lottery method. Sixty-one students, the desired sample size, were chosen from each arm of the designated schools. In each school, the assigned sample size was selected using a simple random technique (lottery method) from the list of students.

### Inclusion and exclusion criteria

The inclusion criterion was adolescents aged 10–19 years. These age cutoff points were chosen based on the definition of adolescents by the World Health Organization (WHO) (25). Students with a younger age (<10 years) or older age (>19 years); who did not give consent to participate in the study; who had chronic diseases such as thyroid, hemolytic disorder; who had previously been diagnosed with a mental disorder, such as depression or bipolar disorder according to DSM-5 (Diagnostic and Statistical Manual of Mental Disorders, Fifth Edition) (26); who received mental health disorder medications; who were currently on iron supplements; or who were pregnant or lactating girls (have different physiological parameters for anemia and serum ferritin) were excluded from participating in this study.

### Sample size calculation

A sample size of 61 participants in each study group was computed with an equal number of cases and controls (ratio of 1:1). The sample size was calculated assuming a mean difference in serum ferritin (8.0 μg/l) between depressed and nondepressed adolescents. This assumption was based on a study showing a mean serum ferritin level of 8.0 μg/l among adolescents in the same area as this study (27). The sample size (61 participants in each group) was determined to detect a 5% difference at an α-value of 0.05 with 80% power.

### Study variables and measures

A questionnaire was developed based on previous studies (18, 21, 28). This questionnaire included data on sociodemographic characteristics, such as age in years, gender (male or female), parental educational levels (<secondary or ≥secondary), mother’s occupational status (housewife or employed), father’s occupational status (skilled worker or laborer), tobacco use (yes or no), and clinical data, such as family history of mental illness. Two medical officers, one male and one female, were trained by the investigators for data collection.

After the participants and their guardians agreed to participate and signed informed consent forms, the selected students were approached, and the questionnaires were completed through face-to-face interviews (by the medical officers). The selected students were informed about the study’s aims. They provided all necessary information, including the voluntary nature of their participation in the research and their right to withdraw at any time without giving a reason. All preventive measures were taken to ensure the privacy and confidentiality of the participants, including the exclusion of personal identifiers during data collection. Depression, weight, height, and serum ferritin were measured using the standard procedures detailed below.

### Weight and height measurements

The students’ weights were measured in kilograms (kg) using standard procedures, which included well-calibrated scales and adjustments to zero before each measurement. Weight was measured to the nearest 100 grams (g). The students stood with minimal movement, with their hands by their sides. Furthermore, shoes and excess clothing were removed. Height was measured to the nearest 0.1 centimeter (cm), with each student standing straight against a wall with their feet together. Finally, the body mass index (BMI) for the age Z-score was determined based on the WHO’s standards (29).

### Blood sample processing

From each student, 3 ml of blood was taken under aseptic conditions for serum ferritin and C-reactive protein (CRP) analyses, using the laboratory methods described in our previously published work (30, 31). The collected blood samples were allowed to clot and then centrifuged and stored at −20°C until analysis. Serum ferritin was measured using an immunofluorescent assay on the IMMULITE 1000 (SIEMENS, CA, USA), following the manufacturer’s instructions. Ferritin concentration is a promising biomarker of iron stores and should be used to diagnose iron deficiency in otherwise apparently healthy individuals (32, 33). Therefore, according to the WHO cutoff point, iron deficiency was defined as serum ferritin less than 12 μg/l with normal CRP (32).

### Statistical analysis

Data were entered into a computer using the IBM Statistical Package for the Social Sciences (SPSS^®^) for Windows, version 22.0 (SPSS Inc., Armonk, New York, United States). The Shapiro–Wilk test was used to evaluate the normality of continuous data, such as age, and serum ferritin, none of which was found to be normally distributed; hence, values are expressed as median (interquartile range [IQR]) and were compared between the two groups by a nonparametric test (Mann–Whitney U test). A chi-square test was used to compare the categorized variables. Spearman’s correlation was performed between the depression scale and serum ferritin levels. Univariate analysis was performed with depression as the dependent variable. In contrast, the independent variables (covariates) included sociodemographic characteristics (age, gender, and parental educational level), family history of mental illness, BMI, and serum ferritin levels. Furthermore, a multivariable model was utilized using variables from the univariate analysis with a p-value of less than 0.20, with backward elimination to adjust for covariates. Adjusted odds ratios (AORs), 95% confidence intervals (CIs), coefficients, and standard errors were calculated as needed. A two-sided p-value of less than 0.05 was considered statistically significant.

## Results

There was no significant difference in the BMI for age Z-score, parent education and occupation, tobacco use, or family history of mental illness between the two groups (61 adolescents in each group). The median age (15.8[IQR 15.3–16.6] years vs. 14.8[IQR 13.8–15.7] years) was significantly higher, and median serum ferritin level (6.3 [IQR 2.9–13.8] μg/l vs. 25.4 [IQR 19.5–37.1] μg/l, p <0.001) was significantly lower in adolescents with depression than in healthy controls. A significant negative correlation was found between the depression scale and serum ferritin levels (r = -0.596, p < 0.001). In addition, female adolescents were more likely to be depressed than their male counterparts (n = 48 [78.7%] vs. n = 13 [21.3%]) (**Table 1**).

**Table 1:** Comparing the sociodemographic variables between adolescents with depression and controls in northern Sudan, 2022.

Variable		Adolescents with depression (n = 61)	Adolescents without depression [control] (n = 61)	p-value
Age, years, median (interquartile range, IQR)		15.8 (15.3–16.6)	14.8 (13.8–15.7)	<0.001
Body mass index for age Z-score, median (IQR)		-0.43 (-1.22–0.45)	-.38 (-1.86–1.03)	0.645
Serum ferritin, μg/l		6.3 (2.9–13.8)	25.4 (19.5–37.1)	<0.001
		Frequency (percentage)		
Gender	Male	13 (21.3)	48 (78.7)	<0.001
	Female	48 (78.7)	13 (21.3)	
Mother’s Education level	≥ secondary	38 (62.3)	32 (52.5)	0.272
	< secondary	23 (37.7)	29 (47.5)	
Father’s education level	≥ secondary	43 (70.5)	34 (55.7)	0.091
	< secondary	18 (29.5)	27 (44.3)	
Mother’s occupation	Employed	4 (6.6)	11 (18.0)	0.054
	Housewife	57 (93.4)	50 (82.0)	
Father’s occupation	Skilled worker	22 (36.1)	26 (42.6)	0.459
	Laborer	39 (63.9)	35 (57.4)	
Tobacco use	No	58 (95.1)	57 (93.4)	0.697
	Yes	3 (4.9)	4 (6.6)	
Family history of mental illness	No	54 (88.5)	56 (91.8)	0.543
	Yes	7 (11.5)	5 (8.2)	
Iron deficiency	No	18 (29.5)	60 (98.4)	<0.001
	Yes	43 (70.5)	1 (1.6)	

In the univariate analysis, while increasing age, being female, and iron deficiency were positively associated with depression, increasing serum ferritin was negatively associated with depression. In addition, depression was not associated with BMI for age Z-score, mother’s education level, father’s education level, mother’s occupation, father’s occupation, tobacco use, or family history of mental illness (**Table 2**).

**Table 2:** Univariate analysis of variables associated with depression among the studied adolescents in northern Sudan, 2022.

Variable		Odds ratio (95% confidence interval)	p-value
Age, years, median (interquartile range, IQR)		1.65 (1.24–2.18)	<0.001
Body mass index for age Z-score, median (IQR)		1.07 (0.84–1.38)	0.551
Serum ferritin, μg/l		0.95 (0.93–98)	0.003
Gender	Male	Reference	
	Female	13.63 (5.73–32.43)	<0.001
Mother’s Education level	≥ secondary	Reference	
	< secondary	0.66 (0.32–1.37)	0.273
Father’s education level	≥ secondary	Reference	
	< secondary	0.52 (0.25–1.11)	0.093
Mother’s occupation	Employed	Reference	
	Housewife	3.13 (0.93–10.46)	0.063
Father’s occupation	Skilled workers	Reference	
	Labourer	1.31 (0.63–2.72)	0.459
Tobacco use	No	Reference	
	Yes	0.73 (0.15–3.44)	0.698
Family history of mental illness	No	Reference	
	Yes	1.45 (0.43–4.85)	0.545
Iron deficiency	No	Reference	
	Yes	143 (18.42–1114.95)	<0.001

In the multivariate analysis, while increasing age (AOR = 1.44, 95% CI 1.05–1.98), being female (AOR = 11.19, 95% CI 4.17–29.99), and iron deficiency (AOR = 76.87, 95% CI 9.08–650.0) were positively associated with depression, increasing serum ferritin was negatively associated with depression (AOR = 0.96, 95% CI 0.94–0.99). In addition, father’s education level and mother’s occupation were not associated with depression (**Table 3**).

**Table 3:** Multivariate analysis of variables associated with depression among the studied adolescents in northern Sudan, 2022.

Variable		Adjusted odds ratio	95% confidence interval	p-value
Age, years, median (interquartile range)		1.44	1.05–1.98	0.023
Serum ferritin, μg/l*		0.96	0.94–0.99	0.017
Gender	Male (reference)			
	Female	11.19	4.17–29.99	<0.001
Father’s education level	≥ secondary (reference)			
	< secondary	1.60	0.60–4.26	0.341
Mother’s occupation	Employed (reference)			
	Housewife	4.07	0.88–18.75	0.071
Iron deficiency*	No (reference)			
	Yes	76.87	9.08–650.0	<0.001

*These variables, serum ferritin level and iron deficiency, were entered into the model individually.

## Discussion

The main findings of this study indicate that older age, female gender, and iron deficiency are significant positive predictors of depression. In contrast, serum ferritin levels were found to be negatively associated with depression. This study reinforces prior research investigating the effects of serum ferritin and iron deficiency on depression in adolescents (9, 16–20, 28). For example, in Egypt, a case-control study including 86 cases and 86 controls reported a significant negative correlation between depression severity and serum ferritin levels among adolescents (21). Unlike hemoglobin levels, serum ferritin and iron deficiency are early indicators of mental disorders (17, 34). For example, in Iran, a case-control study including 67 depressed students and 125 healthy controls reported an association between depression and decreased ferritin levels even before the occurrence of anemia (17). Zarate-Ortiz et al. also reported that adolescent girls with iron deficiency were more prone to experiencing depression (9). Li et al. reported that serum ferritin was significantly lower in late-life depression patients than in healthy older adults (35). It is essential to note that iron deficiency in Sudan is not limited to adolescents, since studies have also reported a high prevalence of iron deficiency in children and pregnant women (36, 37). Chen et al. recommended screening the iron status of children and adolescents with depression and vice versa (28). Due to the key role of iron status in a wide range of mental disorders, including depression, researchers have suggested the use of iron supplements in patients with depression to maintain adequate iron status (12, 38, 39). For instance, Kedir et al. reported in their systematic review and meta-analysis in sub-Saharan Africa that weekly iron–folic acid supplementation proved effective in enhancing serum ferritin and hemoglobin concentrations, resulting in a lower risk of anemia in school-aged children and adolescents compared with a placebo (12).

Researchers have suggested that low serum ferritin, indicating iron deficiency, may contribute to adolescent depression via several mechanisms. Iron is essential for neurotransmitter synthesis (e.g., serotonin and dopamine) and myelination, both of which are implicated in mood regulation (40–42). Deficiencies can disrupt these processes, leading to depression (42). In addition, iron’s role in brain energy metabolism suggests that inadequate levels may impair neuronal function, thereby exacerbating vulnerability to mental health disorders, such as depression (42, 43).

In this study, additional factors were identified as being associated with depression, including increasing age and female gender. Similarly, other studies have reported analogous results (4, 22). For instance, in a survey conducted by Partap et al. across six sub-Saharan African countries, a higher risk of depression was observed among girls, older adolescents, and adolescents experiencing food insecurity (22). The vulnerability of females to depression can be explained in several ways. For example, women and girls lose blood through menstruation during reproductive age, especially those with heavy menstruation (44), resulting in iron deficiency (44, 45). In addition, females have distinct dietary preferences compared to males, such as pica [40], which can lead to iron deficiency [41] and, consequently, an increased risk of depression.

In this study, the association between older adolescents’ age and depression may be explained by the vulnerability of older adolescents to iron deficiency, either less supply or more demand, since puberty triggers a growth spurt. For example, Durà Travé et al., in a study that included 204 healthy adolescents aged 12–14 years, reported that 8.6% of young males (6.6% in the 10–12-year group and 10.4% in the 13–14-year group) and 12.6% of young females (9.5% in the 10–12-year group and 15.5% in the 13–14-year group) were affected by iron deficiency (45).

In this study, other factors, such as BMI, parent education, and occupation, were not found to be associated with depression. In line with these results, other studies have not reported an association between depression and BMI for age Z-score (18). In contrast, other studies have found an association between depression and tobacco use (5, 6), obesity (7–9), and the level of maternal education (10) among adolescents. Such contradictory findings between this study and previous studies should be viewed by researchers as an opportunity to assess their own countries or regions regarding depression and its associated factors, including hematological parameters, to develop localized solutions.

### Implications and future directions

These results have significant implications for public health interventions, underscoring the need for targeted nutritional programs, particularly for adolescent girls, to address iron deficiency and its associated mental health consequences. Furthermore, this study advocates for integrating mental health screening in schools, mainly focusing on at-risk demographics, such as older adolescents and females. Future research should focus on longitudinal studies to unravel the causal pathways between iron status and mental health and explore whether supplementation can mitigate depressive symptoms. It is very important to consider the ongoing war and its devastating impact on food security and mental health, which might worsen children and adolescents’ nutritional status and mental health (46–48).

### Strengths and limitations of the study

To the best of the authors’ knowledge, this is the first study to investigate the association between serum ferritin and iron deficiency and depression in Sudan, especially in an understudied area. It is important to mention that iron deficiency can be prevented with adequate nutritional intake and even intravenous iron infusions, if required (12, 33, 38, 39), and, as a consequence, decreasing depression among adolescents. However, this study has some limitations that need to be mentioned. Sample size and generalizability: The school-based and localized nature of the study, specifically in northern Sudan, may have limited the sample size, thereby affecting the generalizability of the findings. The findings may not be representative of other demographics, including different age groups, cultural backgrounds, or regions within Sudan. Study design: Because this was a case-control study design (fails to establish causality, making it difficult to determine whether low serum ferritin contributes to depression or vice versa) investigated the association between the studied variables at one point in time. A longitudinal study is recommended since the bidirectional association between nutrition and depression remains unclear (15). Confounding variables: There may be other confounding variables that the study did not fully account for that could affect the relationship between ferritin levels, iron deficiency, and depression in adolescents. These factors may include socioeconomic status, nutritional intake, genetic predispositions, or other health conditions that can impact both iron levels and mental health. The study uses the PHQ-9 questionnaire, which is a validated screening tool but not a diagnostic tool or a severity assessment tool. Due to the sample size limitation, the cutoff of >4 for depression may include mild cases. These limitations should be taken into account in future research.

## Conclusion

This study revealed a notable negative association between serum ferritin levels and depression, indicating that iron deficiency is positively associated with depression in adolescents in northern Sudan. These findings imply that adolescents with depression should be assessed for serum ferritin and iron deficiency, and vice versa.

## Data Availability

The raw data supporting the conclusions of this article will be made available by the authors, without undue reservation.
